# Host factor Rab4b mediates internalization and intoxication of 3D4/21 cells by the active subunit of the *Glaesserella parasuis* cytolethal distending toxin via influencing EEA1 expression

**DOI:** 10.3389/fmicb.2025.1660176

**Published:** 2025-10-31

**Authors:** Yiwen Zhang, Zhen Yang, Senyan Du, Qin Zhao, Xiaobo Huang, Rui Wu, Yiping Wang, Qigui Yan, Sanjie Cao, Yiping Wen

**Affiliations:** ^1^Research Center for Swine Diseases, College of Veterinary Medicine, Sichuan Agricultural University, Chengdu, China; ^2^Chongqing Academy of Animal Sciences, Chongqing, China

**Keywords:** *Glaesserella parasuis*, Rab4b, *Gp*CDT, EEA1, vesicle trafficking

## Abstract

**Background:**

The cytolethal distending toxin (CDT), a significant exotoxin, is closely linked to the pathogenicity of *Glaesserella parasui*s (GPS), but its pathogenic not yet fully elucidated. Previously, we identified Rab4b as a potential host factor contributing to the cytotoxicity of *Gp*CDT through a whole-genome CRISPR/Cas9 screen technology, and subsequently confirmed its association with *Gp*CDT cytotoxicity in PK-15 cells.

**Aims:**

In this study, our data first indicated that Rab4b could interact with the active subunit of the *Glaesserella parasuis* cytolethal distending toxin.

**Methods:**

Investigating the relationship between Rab4b and *Gp*CDT subunits as confirmed by coimmunoprecipitation assay. Next, the porcine alveolar macrophage cell line 3D4/21 was used to establish an infected cell model. Using CRISPR/Cas9 gene editing, we established Rab4b and EEA1-expression-deficient 3D4/21 cell lines. 3D4/21 cells, Rab4b-KO cells and EEA1-KO cells were treated with *Gp*CDT. Cell Counting Kit-8 (CCK-8) assay was used to detect cell viability. Western blotting and qRT-PCR were used to measure the expression of related proteins and genes, and cell morphology observation and indirect immunofluorescence were performed to evaluate the *Gp*CDT-mediated cytotoxicity. Then utilise transcriptome sequencing analysis to investigate its specific mechanisms.

**Result:**

In this study, our data first indicated that Rab4b could interact with the active subunit of the *Gp*CDT. Next, we demonstrated that Rab4b also influences *Gp*CDT-induced cytotoxicity and vesicle trafficking in 3D4/21 cells. To investigate the Rab4b-mediated cytotoxicity of *Gp*CDT in 3D4/21 cells, we screened for EEA1, a gene critical in this process, by transcriptome sequencing analysis. 3D4/21 cells exposed to *Gp*CDT exhibit upregulated EEA1 expression, an event that is lost in the absence of Rab4b. Using CRISPR/Cas9 gene editing, we established EEA1 expression-deficient 3D4/21 cell lines that fail to internalize GpCdtB, resulting in resistance to GpCDT-induced toxic effects.

**Conclusions:**

We suggest that Rab4b facilitates the cellular uptake of *Gp*CDTby upregulating EEA1 protein expression, thereby facilitating the vesicular transport of *Gp*CDT in 3D4/21 cells. Our findings may provide new insights into the pathogenicity of *Gp*CDT and lay the experimental foundation for a deeper understanding of the role of Rab4b proteins

## 1 Introduction

*Glaesserella parasuis* (formerly known as *Haemophilus parasuis*) is a commensal microorganism of the upper respiratory tract of swine and the causative agent of *Glässer*’s disease (Costa-Hurtado et al., 2020), which is characterized by fibrinous polyserositis, plasmacytosis, meningitis, and arthritis (Brockmeier et al., 2013; Mao et al., 2023; Ni et al., 2020). Cytolethal distending toxins (*Gp*CDT) are a class of thermally unstable genotoxins produced by Gram-negative pathogens, which are secreted by a variety of bacteria, including *Actinobacillus actinomycetemcomitans*, *Haemophilus ducreyi*, *Escherichia coli*, and *Campylobacter jejuni* (Pons et al., 2019; Scuron et al., 2016). The complete CDT consists of three subunits: CdtA, CdtB, and CdtC, they are encoded by consecutive genes within a single manipulator (Gargi et al., 2012). The CdtA and CdtC subunits work together to form the “B component,” which facilitates the binding and transport of the “catalytically active a component,” CdtB, which then binds and translates into the host cell. This is similar to the classical “A-B” functional structure of many intracellularly acting bacterial exotoxins (Dixon et al., 2015; Pons et al., 2019; Shenker et al., 2016). CDT was the first toxin protein identified in bacteria capable of damaging the nuclear DNA of target cells (Dixon et al., 2015). Crossing the cell membrane and reaching the target cell’s nucleus is a key step in its DNA-damaging action. Like many bacterial toxins, CdtA and CdtC act as binding components, attaching to cholesterol- and sphingomyelin-rich membrane microdomains (also known as lipid rafts), thereby enhancing the delivery of CdtB into the cell (Chen et al., 2020; Dixon et al., 2015; Yeh et al., 2020). The host cell absorbs and binds *Gp*CDT in the extracellular environment, which triggers the DNA damage response and stops the cell cycle from progressing, eventually causing DNA damage (Boesze-Battaglia et al., 2020; Huhn et al., 2022; Kailoo et al., 2021).

The Rab protein family is the largest group of small GTPases within the Ras superfamily, comprising over 60 members in the human genome (Spano and Galan, 2018). Multiple Rabs can exist on a single intracellular compartment, each occupying its own unique “microstructural domain” (Brumell and Scidmore, 2007; Jordens et al., 2005). A distinct Rab protein mediates each step of the endocytosis pathway. Activated Rab5 co-mediates with Rab4 the transport of extracellular macromolecules from the plasma membrane to early endosomes, acting as a marker for these endosomes (Kalin et al., 2016; Somsel Rodman and Wandinger-Ness, 2000). Rab4b, as a subtype of Rab4, is a key protein involved in vesicle trafficking and is mainly localised in lattice-encapsulated vesicles, early endosomes, and circulating endosomes and is an important regulator of cellular endocytosis and cycling processes (He et al., 2002; Krawczyk et al., 2007; Pereira-Leal and Seabra, 2001; Perrin et al., 2013). In 1999, scholars first examined the presence of Rab4b protein in human umbilical vein endothelial cells. Subsequently, Rab4b was also detected to be upregulated in hepatocellular carcinoma and intrahepatic cholangiocarcinoma (Perrin et al., 2013). Rab4b was also detected in mouse adipocytes (3T3-L1), lung, and myocardial tissue (Kaddai et al., 2009).

Early endosomal antigen 1 (EEA1) is a cytosolic protein that specifically binds to early endosomal membranes, where it plays a crucial role in the tethering process leading to homotypic endosome fusion (Bergeland et al., 2008). EEA1 is a long coiled-coil homodimer with 17–20% homology to myosin. It contains calmodulin-binding IQ (isoleucine and glutamine) motifs associated with these proteins, which are involved in cellular uptake functions, promote the formation of vesicles encapsulating toxin proteins (Mu et al., 1995), and control the transport of vesicles to the early endosomes and then fuse with early endosomes (Fouraux et al., 2004; Mishra et al., 2010; Rubino et al., 2000). Rab5 can regulate the production of lattice protein-encapsulated vesicles, and EEA1, an effector protein of Rab5, has been shown to undergo a conformational change on vesicles upon binding to Rab5-GTP and to provide the mechanical force necessary to disengage vesicles from the cell membrane and bring them closer to the early endosomes or other vesicles (Adams and Wayne Vogl, 2017; Mills et al., 1998; Simonsen et al., 1998; Stenmark et al., 1996). The C-terminal FYVE structural domain of the EEA1 protein binds to abundant PI3P on early endosomes and promotes vesicle fusion with early endosomes (Rubino et al., 2000). EEA1 regulates numerous biological events through its effects on cellular uptake functions.

The CRISPR/Cas9 gene editing technology can induce random or targeted gene mutations, leading to the loss of protein function. The affected regions include the coding and non-coding regions of the gene. CRISPR/Cas9 technology has become the most extensively examined gene editing technology in recent years due to its simple design, low cost, high efficiency, and ease of operation, which can also achieve simultaneous editing of multiple loci. It can also be carried out without using plasmids, thereby saving the trouble caused by plasmids. CRISPR/Cas9 has shown great potential in studying genes and genomic functions in microorganisms, plants, animals, and humans (Bergeland et al., 2008). CRISPR/Cas9 can systematically screen out host proteins that directly or indirectly participate in the cytotoxicity of *Gp*CDT, including toxin receptors on the cell membrane and interacting/non-interacting proteins within the cell (Hesping and Boddey, 2024; Hu and Wang, 2024; Schoellkopf et al., 2022). In the early stage, we utilized the CRISPR/Cas9 technology to identify host proteins involved in the cytotoxicity of *Gp*CDT. We successfully identified multiple host proteins, including the host protein Rab4b. Considering Rab4b’s significance in cell membrane transport, we investigated its potential involvement in the cytotoxicity of 3D4/21, which *Gp*CDT produces.

## 2 Materials and methods

### 2.1 Cell lines, plasmids, and antibodies

3D4/21 cells were cultured in a complete medium of RPMI-1640 (Gibco, Carlsbad, CA, United States). Human embryo kidney (HEK-293T) cells were grown in DMEM (Gibco, Carlsbad, CA, United States). Both cells required the addition of 10% fetal bovine serum (Gibco, Carlsbad, CA, United States). They were subcultured upon reaching 90% confluence and incubated in a 37 °C incubator containing 5% CO_2_. The bacterial strains and plasmids used in this study are listed in Table 1. All strains were grown in broth with shaking at 180 rpm at 37 °C.

**Table 1:** Bacterial strains and plasmids used in this study.

Strain or plasmid	Relevant characteristics	Source
*BL21(DE3)*	*E. coli* str. B F^−^ ompT gal dcm lon hsdSB(rB^−^mB^−^) λ (DE3 [lacI lacUV5-T7p07 ind1 sam7 nin5]) [malB+]K-12(λS)	Biomed
pET-*cdtA*	A 624 bp cdtA CDS in pET-32a (+)	Laboratory collection
pET-*cdtB*	A 768 bp cdtB CDS in pET-32a (+)	Laboratory collection
pET-*cdtC*	A 471 bp cdtC CDS in pET-32a (+)	Laboratory collection
pMD2.G	Lentivirus envelope plasmid	Laboratory collection
pSPAX2	Lentivirus envelope plasmid	Laboratory collection
pLentiCRISPR V2	sgRNA*^a^* carrier plasmid	Laboratory collection
pEGFP-N1-Rab4b	Overexpression plasmid	Laboratory collection
pcDNA-3.1-Flag-Rab4b	Overexpression plasmid	Laboratory collection

Antibodies for caspase-3 (ab32351), EEA1 (ab109110), Rab5 (ab218624), and RCAS1 (ab200348) were purchased from Abcam (Cambridge, Cambridgeshire, Britain). Antibodies for Flag (66008-4-Ig), and His (66005-1-IG) were from Proteintech Group (Wuhan, Hubei, China). Na⁺/K⁺-ATPase (P06685) and γH2AX (9718T) were purchased from Cell Signaling Technology (Danver, MA, United States). β-actin (High Dilution) (AC026) and the secondary antibodies including HRP Goat Anti-Mouse IgG (H + L) (AS003), HRP Goat Anti-Rabbit IgG (H + L) (AS014), Alexa Fluor 488-conjugated Goat anti-Rabbit IgG (H + L) (AS053) and Alexa Fluor 594-conjugated Goat anti-Rabbit IgG (H + L) (AS039) were bought from Abclonal (Wuhan, Hubei, China). Cell Plasma Membrane Staining Kit with DiI (Red Fluorescence) (C1991S) was bought from Beyotime (Shanghai, China).

A previous report has been published on the production of Rab4b antiserum. Simply put, Rab4b antiserum was produced by immunizing mice with the C-terminal peptide of Rab4b conjugated to KLH (Zhang et al., 2024).

### 2.2 Preparation of recombinant *Gp*CDT protein and mouse antiserum

Construction and expression of plasmids containing *cdtA*, *cdtB*, and *cdtC* genes have been reported previously. In brief, the constructed plasmids were transformed into Rosetta (DE3) pLysS. Recombinant clones were induced with 0.2 mM IPTG for 12 h at 25 °C to achieve optimal expression. These three His-tag fusion recombinant proteins were purified by Ni affinity chromatography (Bio-Rad, Hercules, CA, United States) and then confirmed by SDS-PAGE electrophoresis. In this study, the three subunits were reconstituted as a holotoxin at 4 °C overnight.

The expression of anti-*Gp*CdtA, anti-*Gp*CdtB, and anti-*Gp*CdtC antisera was also as previously described. Each mouse was immunized subcutaneously on days 0, 14, and 21 with 0.1 mg of *Gp*CdtA, *Gp*CdtB, or *Gp*CdtC (200 μL) and 20 μL of water adjuvant Montanide Gel 01 (SEPPIC, France). Twenty-eight days later, blood was collected from the mice and left at 4 °C overnight. The serum was then collected and stored in the refrigerator (Yang et al., 2023).

### 2.3 Coimmunoprecipitation assay

HEK-293T cells at 50% confluence in 6-well plates were cotransfected with pcDNA-3.1-Flag-Rab4b. At 36 h post-transfection, the cells were incubated with 10 μg/mL *Gp*CDT for 30 min. Then, coimmunoprecipitation assays were performed using Protein A/G Magnetic Beads (MedChemExpress, United States). Western blot analysis was performed using antisera against *Gp*CdtA, *Gp*CdtB, and *Gp*CdtC.

To analyze the interaction between *Gp*CDT subunits and the host protein Rab4b, HEK-293T cells were seeded into 6-well culture plates and transfected with the corresponding expression plasmid. Transfected cells were harvested at 48 h post-transfection and lysed in cell lysis buffer containing one mM protease inhibitor. After centrifugation at 14,000 g for 10 min, the lysate supernatant was incubated with 2.5 μg of His-*Gp*CDT subunits for 4 h with gentle rocking at 4 °C. It was then incubated overnight with mouse monoclonal antibodies (mAbs) against the Flag or His tag, also with gentle rocking at 4 °C. Protein A/G Magnetic Beads washed with cell lysate were added to the supernatants and incubated with gentle rocking for 4 h at 4 °C. The beads were washed four times with cold cell lysate and then boiled in SDS loading buffer for 10 min, followed by Western blot analysis.

### 2.4 Rab4b and EEA1 knockout

The Rab4b and EEA1 small guide RNA (sgRNA) were inserted into the lentiCRISPR-V2 plasmid (Table 2). Using Lipofectamine 3000, the latter were transfected into HEK-293T cells. After a 40-h incubation, the lentivirus-containing supernatants were harvested. 3D42/1 cells were infected with the harvested lentiviruses by incubation for 24 h, and 8 μg/mL puromycin was added to the screen, followed by Rab4b and EEA1 knockout screening for stable Rab4b and EEA1 knockout (“Rab4b-KO” and “EEA1-KO”) cells.

**Table 2:** Primers used in this study.

Gene	Primer direction	Sequence (5′–3′)	Size (bp)
Rab4b-sgRNA	Forward	CACCGTGACGCGGAGTTATTACCG	24
	Reverse	AAACCGGTAATAACTCCGCGTCAC	
Rab4b-KO	Forward	CACAATCGGCGTGGAGTT	176
	Reverse	AGTTGTAAGTCTCCCGGCTGT	
EEA1	Forward	AACGAGGCGAAACGTACCAT	140
	Reverse	ACTGCGATTTCCCCCGTAAG	
EEA1-sgRNA	Forward	CACCTTACATGAATACCAACCACG	24
	Reverse	AAACCGTGGTTGGTATTCATGTAA	
EEA1-KO	Forward	GATGACCGCATTAAACGAAAAC	112
	Reverse	AGCACTTTTCTCAAGACTTCGG	

### 2.5 Western blotting

After cells were infected with *Gp*CDT, they were lysed in RIPA buffer (Proteintech, Wuhan, China) and centrifuged. The supernatant was then collected for Western blotting. Samples were then moved to PVDF membranes after being separated on 12.5% SDS-PAGE. The membranes were blocked with 5% nonfat dry milk before the primary antibody was incubated. Then, the membranes were treated with a second antibody after being washed with PBS. The Clarity Max Enhanced Chemiluminescence (ECL) (Bio-Rad, Hercules, CA, United States) was added to membranes and captured by ImageJ software (National Institutes of Health).

### 2.6 Quantitative real-time PCR

Using the UNIQ-10 column total RNA purification kit (Sangon, China), RNA was extracted from 3D4/21 cells that had been treated with *Gp*CDT. The PrimeScript TM RT kit with gDNA Eraser (Takara, Japan) was used to conduct a two-step RT-PCR. Using SYBR premix EX Taq^™^ II (Tli RNaseH Plus; Takara, Japan), transcripts were analyzed by qRT-PCR. The 2^−∆∆CT^ method was used to quantify gene expression, and results are presented relative to expression of β-actin. Table 2 lists the primer sequences that were employed.

### 2.7 Microscopy imaging

Cells were seeded in 6-well tissue culture plates (5 × 10^5^ cells per well). After *Gp*CDT exposure for 48 h, static bright field images of cells were captured using light microscopy (Olympus America, Center Valley, PA).

### 2.8 Cell viability assay

For detecting cell viability, cells were pre-seeded into 96-well plates and treated with *Gp*CDT (10 μg/mL) for 0, 12, 24, 36, 48, and 60 h. A routine CCK-8 assay was used to examine cell viability according to the manufacturer’s protocol.

### 2.9 Indirect immunofluorescence

In 6-well plates, cells were grown to around 90% confluence. Purified *Gp*CDT (10 μg/mL) was then added to each well, and the cells were incubated for 12 and 24 h at 37 °C. Following three PBS washes, the cells were fixed with 4% paraformaldehyde for 15 min before being rinsed with PBS once more. After that, cells were blocked for an hour at 37 °C in a BSA (3%) solution. Anti-H2AX primary antibody (1:1,000; Abcam, MA, United States) was incubated with cells overnight at 4 °C. Samples were then incubated at 37 °C for 1 h in the dark with fluorescein isothiocyanate (FITC)-conjugated goat anti-mouse IgG (Proteintech, Beijing, China). To identify the nuclei, DAPI (Beyotime, Shanghai, China) was utilized.

Trafficking studies were performed as follows. Cells were inoculated in 6-well plates and incubated with 10 μg/mL *Gp*CDT for 30 min at 4 °C to promote binding, followed by incubation at 37 °C for 45 min to stimulate uptake. Cells were subsequently immunostained for *Gp*CdtB and intracellular markers.

### 2.10 Statistical analysis

Statistical analyses were performed using GraphPad Prism version 8.0 (CA, United States). Statistical significance was assessed using Student’s *t*-test, one-way ANOVA, or two-way ANOVA. Significant differences between groups are indicated by ^*^p < 0.05, ^**^p < 0.01, ^***^p < 0.001, and ^****^p < 0.0001.

## 3 Results

### 3.1 Interaction analysis of Rab4b and *Gp*CDT subunits

**Figure 1:**
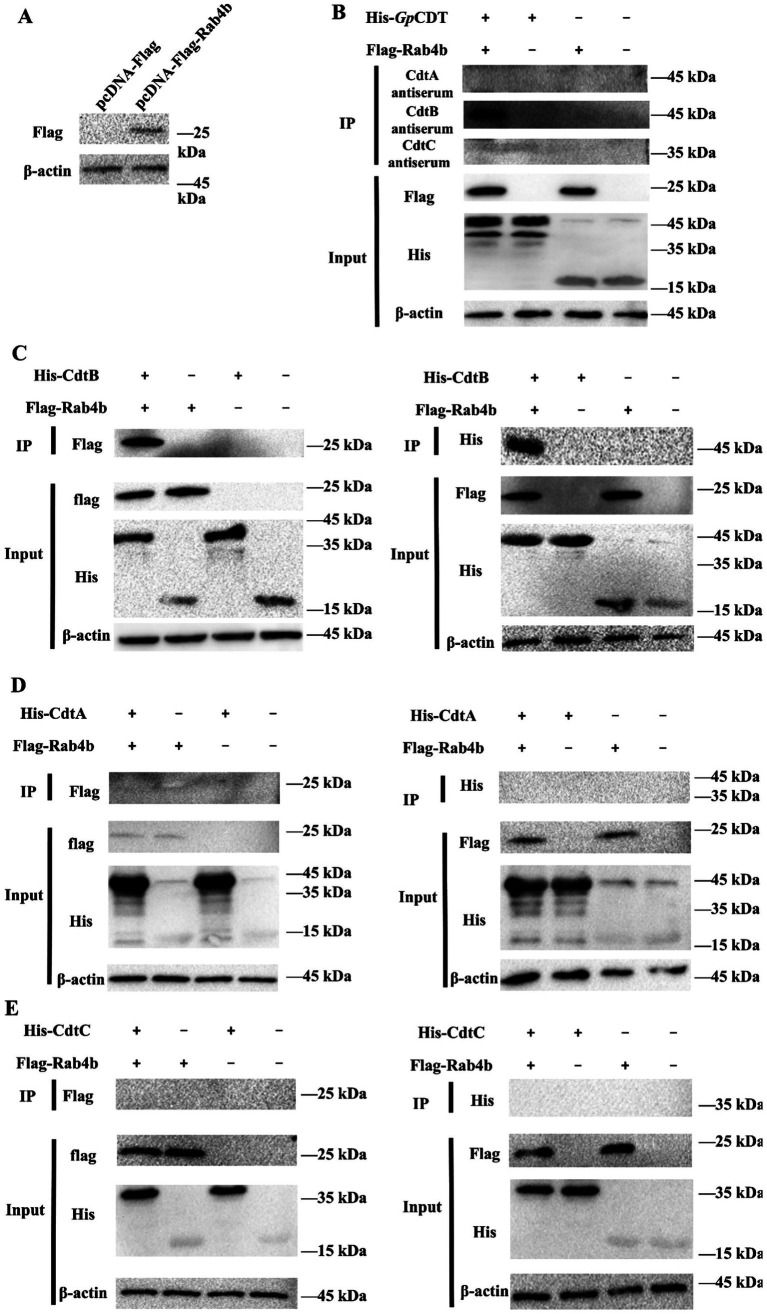
The interaction of Rab4b and CTD subunits was detected by coimmunoprecipitation. **(A)** Expression of the Rab4b gene on HEK-293T cells was detected by Western blot. **(B)** Immunoblot of host factor Rab4b recombinant protein from transfected HEK 293T cells and His-*Gp*CDT protein precipitated using anti-*Gp*CdtA, anti-*Gp*CdtB, and anti-*Gp*CdtC antisera. **(C–E)** Interactions between host factor Rab4b and His-*Gp*CdtA were analyzed by using anti-Flag mAb or anti-His mAb.

We previously demonstrated that Rab4b has the potential for a direct interaction with *Gp*CDT (Zhang et al., 2024). The *Gp*CDT subunits have molecular masses of approximately 42 kDa (*Gp*CdtA), 47 kDa (*Gp*CdtB), and 36 kDa (*Gp*CdtC) (Yang et al., 2023). To further understand how Rab4b controls sorting events in the early endosomes of the *Gp*CDT subunit, the present study aimed to investigate the possible role of Rab4b in this process using a coimmunoprecipitation assay. HEK-293T cells were transfected with expression plasmids to overexpress Flag-Rab4b. The expression of this construct was confirmed by Western blotting (Figure 1A). In addition, co-IP was performed with anti-*Gp*CdtA antiserum, anti-*Gp*CdtB antiserum, anti-*Gp*CdtC antiserum, anti-Flag mAb, or anti-His mAb to capture protein complexes. As shown in Figure 1B, anti-*Gp*CdtA and anti-*Gp*CdtC antisera did not result in imprint bands, but *Gp*CdtB could be observed in the IP samples by using anti-*Gp*CdtB antisera. In the next series of experiments, we incubated Rab4b directly with the *Gp*CdtA, *Gp*CdtB, and *Gp*CdtC subunits. Coimmunoprecipitations, performed using anti-His and anti-Flag mAb, showed that Rab4b coimmunoprecipitated with *Gp*CdtB (Figure 1C) but not with *Gp*CdtA and *Gp*CdtC (Figures 1D,E). These results suggested that Rab4b can interact with the *Gp*CdtB.

### 3.2 Rab4b influences *Gp*CDT from *Glaesserella parasuis*-induced cytotoxicity and vesicle trafficking in 3D4/21 cells

**Figure 2:**
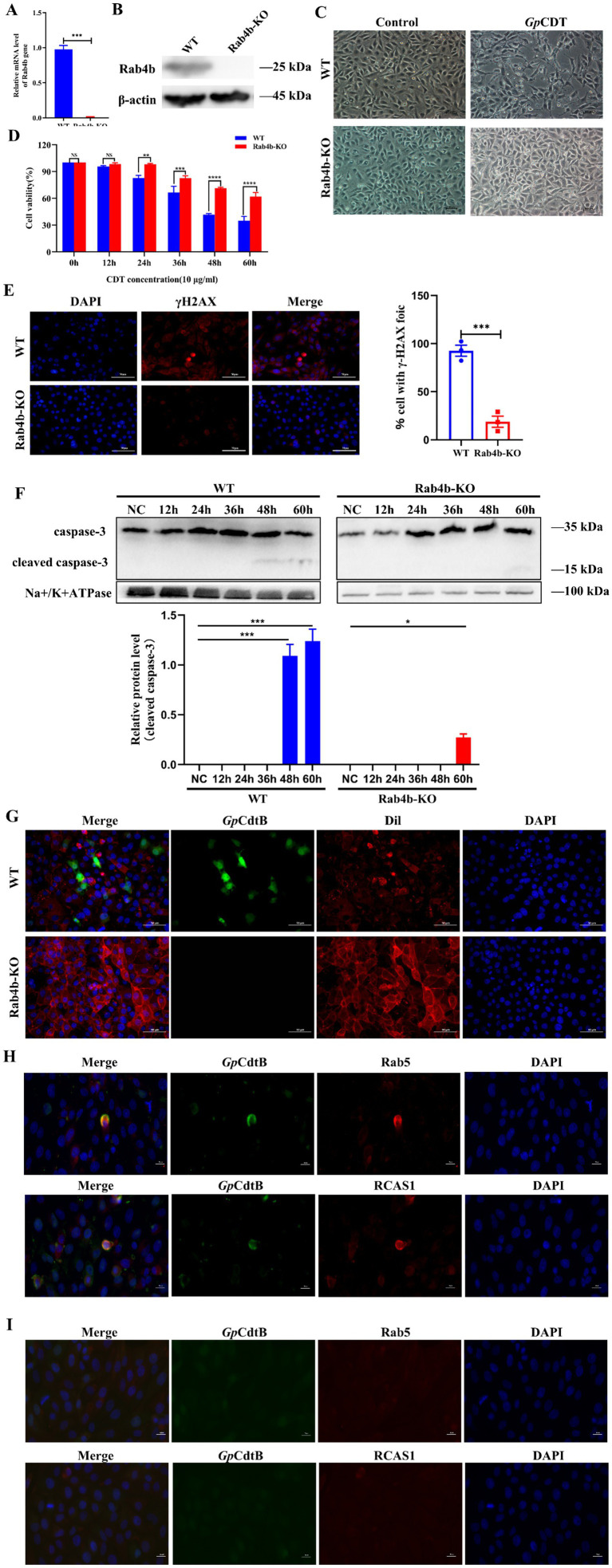
Knockout of Rab4b weakened *Gp*CDT-induced cytotoxicity and vesicle trafficking in 3D4/21 cells. **(A)** Detection of Rab4b gene mRNA level by qRT-PCR. **(B)** Western blot analysis comparing Rab4b protein expression level in3D4/21 and Rab4b-KO cells. **(C)** Typical morphology of 3D4/21 cells and Rab4b-KO cells treated with 10 μg/mL *Gp*CDT. Scale bar 100 μm. **(D)** Cell viability after 10 μg/mL *Gp*CDT exposure for varying durations (0, 12, 24, 36, 48, and 60 h) (* means *p* < 0.05, ** means *p* < 0.01, *** means *p* < 0.001, **** means *p* < 0.001, and ns means *p* > 0.05). **(E)** The DNA damage signature, as indicated by γH2AX, was detected using immunofluorescence microscopy with a bar graph quantifying γH2AX foci. Scale bar 50 μm. **(G)** The entry of *Gp*CDT into 3D4/21 cells and Rab4b-KO cells was detected by indirect immunofluorescence: *Gp*CDT B (green) and Dil (red). Scale bar 50 μm. **(H,I)** For the trafficking study, the indirect immunofluorescence results for the 3D4/21 and Rab4b-KO cells were obtained after treatment with *Gp*CDT. Green fluorescence indicates the *Gp*CdtB, and red fluorescence indicates the organelle marker. Rab5 is an early endosomal marker, and RCAS1 is a marker of the Golgi apparatus. Scale bar 20 μm.

We used the CRISPR/Cas9 gene editing method to knock out Rab4b (see the Methods section). qRT-PCR and Western blotting results confirmed that Rab4b mRNA (Figure 2A) and protein (Figure 2B) were knocked out entirely in Rab4b-KO cells. Next, we examined the morphology of 3D4/21 cells and Rab4b-KO cells after treatment with *Gp*CDT. Knockout of Rab4b resulted in significantly exhibited less cell distention after 48 h of *Gp*CDT treatment (Figure 2C). Furthermore, Rab4b-KO cells demonstrated a considerable improvement in cell viability after *Gp*CDT treatment (Figure 2D). Moreover, as shown in Figure 2E, *Gp*CDT treatments for 24 h increased the number of gH2AX foci, a marker for DNA damage signaling, in 3D4/21 cells, when compared with Rab4b-KO cells. Moreover, in control 3D4/21 cells, *Gp*CDT treatment induced caspase-3 cleavages (Figure 2F). These results indicate that Rab4b is necessary for *Gp*CDT-induced cytotoxicity against the 3D4/21 cells.

Early endosomes and sorting endosomes, which are located where the endocytic and exocytic pathways converge, have been important hubs for membrane trafficking. We first demonstrated that in the presence of Rab4b, 3D4/21 cells took up *Gp*CDT inwards (Figure 2G). Once internalized, CdtB first reaches the early endosomes. Rab4b, located in the early endosomes, then exerts its function of controlling early endosome sorting, regulating the translocation of internalized *Gp*CdtB to the Golgi apparatus and endoplasmic reticulum (ER) in a retrograde manner in several cell types. 3D4/21 cells treated with *Gp*CDT display fluorescence of *Gp*CdtB (green), which co-localises with early lysosomes and the Golgi apparatus (red). This indicates that CdtB transport from early endosomes (Rab5+) to the Golgi apparatus (RCAS1+) also occurs in 3D4/21 cells (Figure 2H). Noteworthy is that we examined the vesicle trafficking of *Gp*CDT in Rab4b-KO cells to investigate the impact of Rab4b on this process. The results revealed that in Rab4b-KO cells, the red fluorescence of *Gp*CdtB did not colocalize with the green fluorescence of Rab5 and RCAS1 (Figure 2I), indicating that *Gp*CDT failed to reach the early endosome after Rab4b knockout. In summary, these findings show that Rab4b influences vesicle trafficking in *Gp*CDT.

### 3.3 Using comparative transcriptomic analysis, EEA1 was found to be a crucial gene for Rab4b-mediated internalization and intoxication of 3D4/21 cells by the active subunit of the *Glaesserella parasuis* cytolethal distending toxin

**Figure 3:**
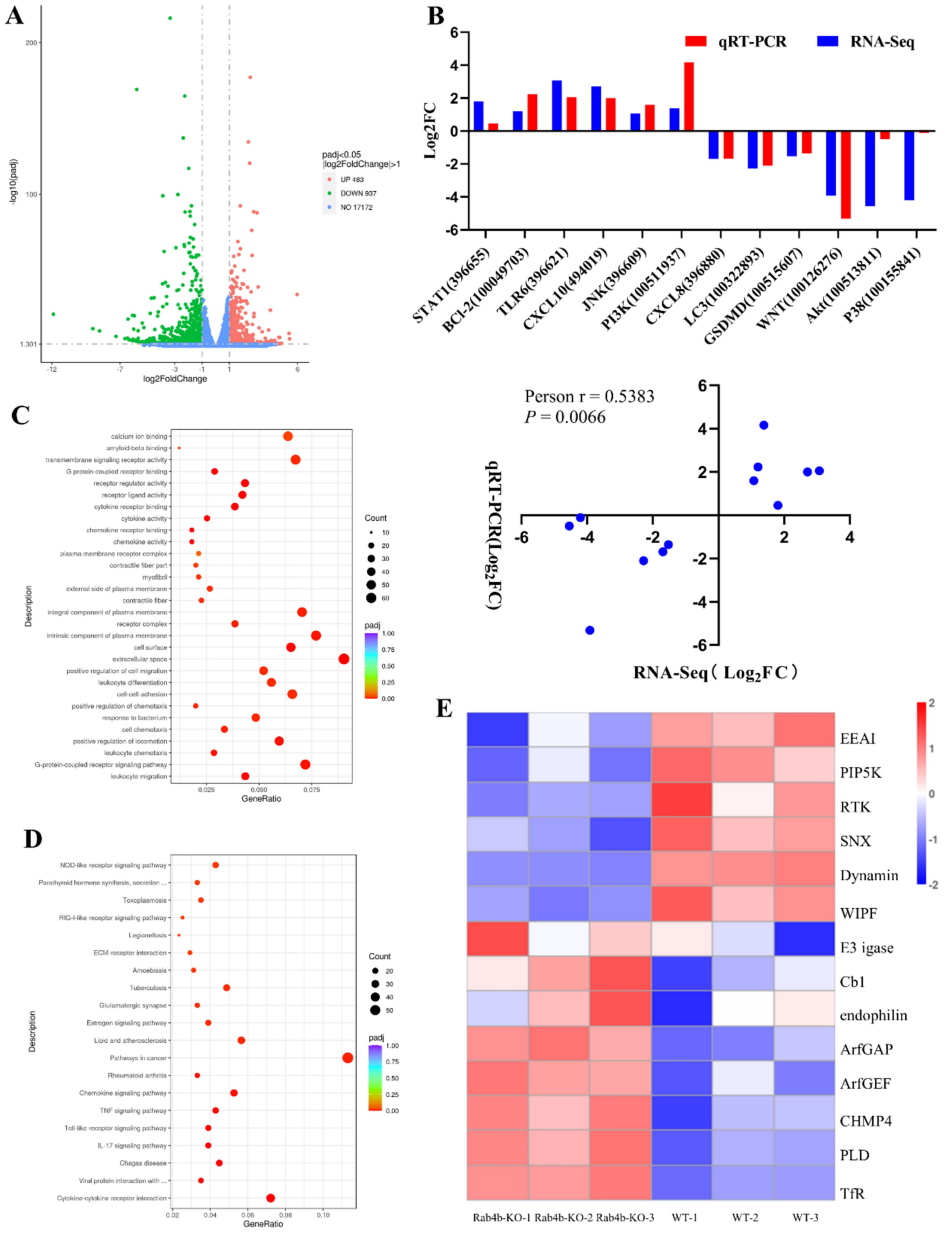
Comparative transcriptomic analysis (Rab4b-KO vs. 3D4/21 Cells). **(A)** The differentially expressed genes (DEGs) in Rab4b-KO and 3D42/1 cells infected with *Gp*CDT were analyzed via volcano plots. Red dots indicate DEGs that are upregulated, green dots indicate downregulated DEGs, and blue dots indicate genes that do not significantly differ. **(B)** DEGs were selected randomly for qRT-PCR analysis, and the expression levels of those DEGs were estimated using the 2^−∆∆CT^ method. The correlation between DEG expression levels, as determined by RNA sequencing and qRT-PCR, was analyzed using Pearson’s correlation analysis. **(C)** Bubble map of the top 20 most enriched GO terms. **(D)** Histograms of the top 20 most enriched signaling pathways. **(E)** A heat map that shows the expression of endocytosis pathway-related genes.

To explore the role of Rab4b after *Gp*CDT treatment, we conducted a comparative transcriptomic study on Rab4b-KO cells and 3D42/1 cells incubated with purified *Gp*CDT for 12 h. Following quality control, all samples had Q20 and Q30 percentages of clean data greater than 97 and 93%, respectively. Each sample’s clean readings had a GC content ranging from 51.63 to 53.34% (Supplementary Tables S1). Transcriptome analysis revealed 1,420 differentially expressed genes (DEGs) after 12 h in the Rab4b-KO group compared with the 3D4/21 control group. Of these genes, 483 were upregulated and 937 were downregulated (Figure 3A). Six genes with elevated transcription levels and six with downregulated transcription levels were arbitrarily chosen for relative fluorescence measurement to corroborate the transcriptome sequencing results (Supplementary Tables S2). As shown in Figure 3B, the expression of most of the selected DEGs generated by RNA sequencing was consistent with the levels obtained using qRT-PCR. In addition, the correlation between the expression levels of the selected DEGs obtained using RNA sequencing and qRT-PCR was analyzed by calculating Pearson’s correlation coefficient. The results revealed that Pearson’s correlation coefficient was 0.3331, confirming that the data generated by RNA sequencing were reliable.

KEGG pathway analysis and GO categorization were applied to the DEGs. According to the GO analysis, the most prevalent categories were leukocyte migration, the G-protein-coupled receptor signaling pathway, the extracellular space, the cell surface, and chemokine activity (Figure 3C). KEGG analysis revealed that the significantly enriched pathways were associated with cytokines and cytokine receptors and pathways involved in lipid metabolism and atherosclerosis (Figure 3D). GO and KEGG enrichment analyses suggested that Rab4b influences several biological functions in 3D4/21 cells infected with *Gp*CDT. Rab4b is associated with the endocytosis pathway in the KEGG signaling pathway, which is closely related to cell vesicle trafficking. Fourteen DEGs were identified in the endocytosis pathway (Figure 3E), and only EEA1, like Rab4b, is located in the early endosomes, which can mediate early endosome fusion, as well as the capture of vesicles derived from clathrin-coated pits by early endosomes. EEA1 may be an important protein involved in Rab4b-mediated *Gp*CDT cytotoxicity and vesicle trafficking.

### 3.4 After *Gp*CDT treatment, Rab4b can upregulate the expression of EEA1 in 3D4/21 cells

**Figure 4:**
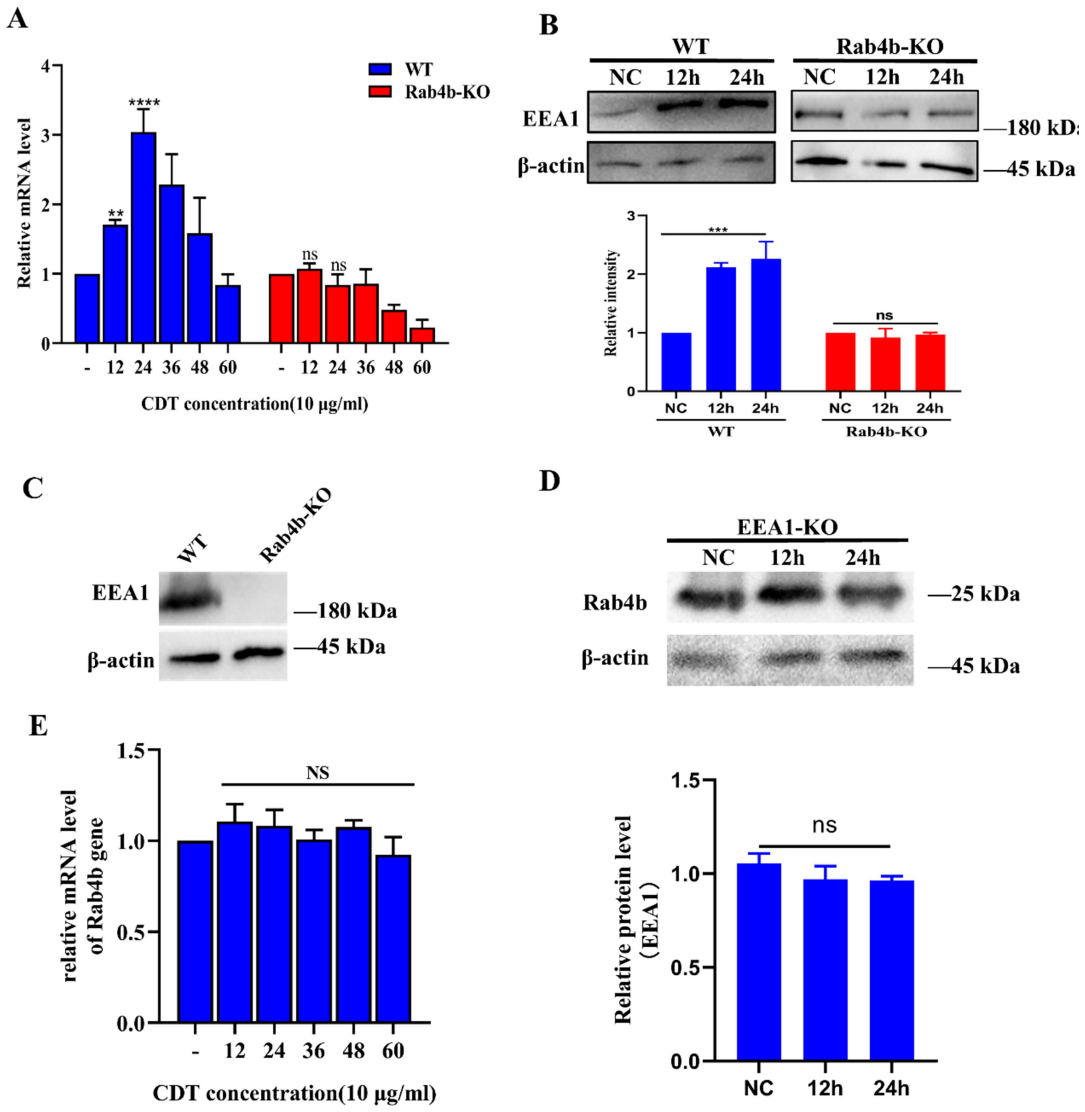
Effect of Rab4b on EEA1 expression in 3D4/21 cells after treatment with *Gp*CDT. **(A)** The mRNA level of EEA1 in *Gp*CDT-treated 3D4/21 and Rab4b-KO cells was measured via qRT-PCR (* means *p* < 0.05, ** means *p* < 0.01, *** means *p* < 0.001, **** means *p* < 0.001, and ns means *p* > 0.05). **(B)** The protein expression level of EEA1 in *Gp*CDT-treated 3D4/21 and Rab4b-KO cells was analyzed by Western blotting. **(C)** The efficiency of EEA1 protein knockout was confirmed by Western blot analysis. **(D)** The expression level of the Rab4b protein in *Gp*CDT-treated EEA1-KO cells was analyzed by Western blotting. **(E)** Detect the mRNA level of Rab4b by qRT-PCR in EEA1-KO cells after *Gp*CDT treatment (* means *p* < 0.05, ** means *p* < 0.01, *** means *p* < 0.001, **** means *p* < 0.001, and ns means *p* > 0.05).

In this study, transcriptomic sequencing revealed that the expression level of EEA1 changed significantly after 12 h of cell infection with *Gp*CDT. The expression levels of EEA1 in 3D4/21 and Rab4b-KO cells, as detected by qRT-PCR and Western blot, after *Gp*CDT treatment. The results showed that the mRNA level of EEA1 increased after *Gp*CDT treatment in WT cells. After the elimination of Rab4b, the mRNA level of EEA1 did not change significantly (Figure 4A). Western blotting results also revealed that the EEA1 protein level increased after 24 h of treatment of 3D4/21 cells with *Gp*CDT, whereas the EEA1 protein level did not change significantly after Rab4b knockout (Figure 4B).

The EEA1 knockout cells were subsequently constructed via CRISPR/Cas9 technology. The western blot results showed that the expression level of the EEA1 protein in EEA1-KO cells was knocked out compared with WT cells, indicating that the EEA1 knockout (“EEA1-KO”) cells were successfully constructed (Figure 4C). Subsequently, using Western blotting and qRT-PCR, the Rab4b mRNA levels in EEA1-KO cells treated with *Gp*CDT were determined. These results confirmed that when EEA1 was knocked out, Rab4b mRNA and protein levels did not alter considerably (Figures 4D,E). These findings suggested that in *Gp*CDT-treated cells, EEA1 did not affect Rab4b transcription levels.

### 3.5 The effect of EEA1 on the Rab4b mediates internalization and intoxication of 3D4/21 cells by the *Gp*CDT

**Figure 5:**
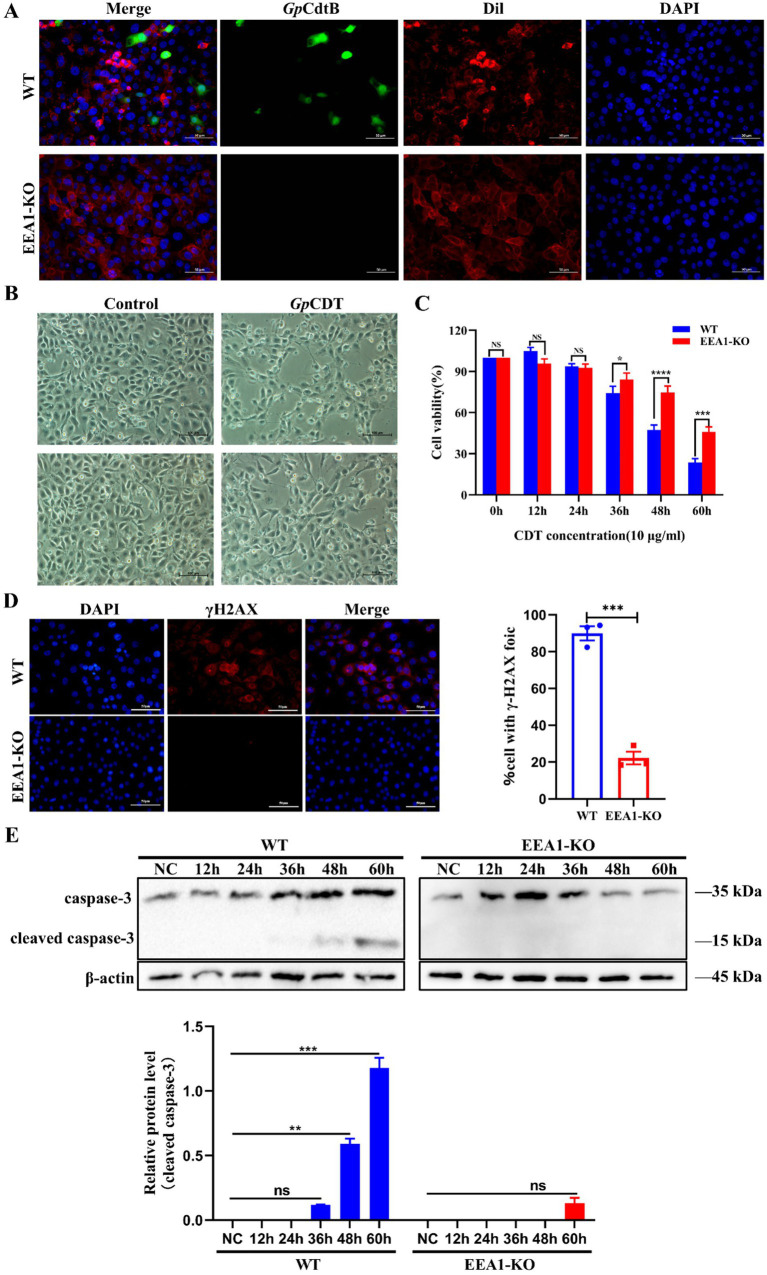
In 3D4/21 cells, the Rab4b-mediated cytotoxicity and vesicle trafficking of *Gp*CDT can be influenced by EEA1. **(A)** The entry of *Gp*CDT into 3D4/21 cells and EEA1-KO cells was detected by indirect immunofluorescence. *Gp*CdtB (green) and Dil (red). Scale bar 50 μm. **(B)** Observed the typical morphology of 3D4/21 cells and EEA1-KO cells after *Gp*CDT treatment for 48 h. Scale bar 100 μm. **(C)** CCK-8 was used to determine the survival rates of 3D4/21 and EEA1-KO cells treated with 10 μg/mL *Gp*CDT at different time points (* means *p* < 0.05, ** means *p* < 0.01, *** means *p* < 0.001, **** means *p* < 0.001, and ns means *p* > 0.05). **(D)** 3D4/21 cells and EEA1-KO cells cells were exposed to G*p*CDT for 24 h, and analyzed by immunofluorescence microscopy with antibodies directed against γH2AX (red) and DAPI (blue), alongside a bar graph showing the percentage of cells with γH2AX foci. Scale bar 50 μm. **(E)** Cleaved caspase-3 levels were detected in 3D4/21 and EEA1-KO cells treated with *Gp*CDT using Western blotting (*n* = 3).

It has been confirmed that knockout of Rab4b can inhibit the expression of the EEA1 protein after *Gp*CDT treatment. The following steps were taken to confirm whether the EEA1 protein influences Rab4b-mediated *Gp*CDT-induced cytotoxicity and vesicle trafficking in 3D4/21 cells. Using indirect immunofluorescence, we were able to identify the fluorescence of *Gp*CdtB and get insight into the uptake of *Gp*CDT by cells following EEA1 deletion. The findings showed that *Gp*CdtB fluorescence was not seen in EEA1-KO cells (Figure 5A). Next, observe the morphological changes that occur as a result of exposure to *Gp*CDT. EEA1-KO cells were more resistant to *Gp*CDT and exhibited less cell distention (Figure 5B). Meanwhile, the study of the EEA1 effect on cell survival rate showed that the survival rate of EEA1-KO cells treated with *Gp*CDT was greater than that of WT cells (Figure 5C). Similarly, we demonstrated that *Gp*CDT reduced the number of γH2AX foci in EEA1-KO cells compared to 3D4/21 cells, a marker for DNA damage signaling (Figure 5D). Western blotting was used to detect the activation level of caspase-3, a key apoptotic factor, after *Gp*CDT treatment. The results showed that the cleaved caspase-3 bands appeared at 48 h after *Gp*CDT treatment, while the caspase-3 cleavage bands appeared at 60 h in EEA1-KO cells (Figure 5E). Overall, the experimental data support our hypothesis that EEA1 affects the Rab4b-mediated internalization and intoxication of 3D4/21 cells by the *Gp*CDT.

## 4 Discussion

The present study investigated the role of Rab4b in mitigating the toxic effects of *Gp*CDT on cells by modulating the vesicle trafficking process of *Gp*CDT and identified key genes involved in this process through transcriptome sequencing analysis. The following are our work’s three main conclusions: (i) Rab4b interacts with the active subunit of *Gp*CDT and influences *Gp*CDT-induced cytotoxicity in 3D4/21 cells; (ii) Rab4b promotes the uptake of *Gp*CDT by cells through the upregulation of EEA1 protein expression and promotes subsequent vesicle trafficking.

Existing studies have reported that Rab4b is involved in various intracellular functions. Antigen-presenting cells APC enhance their antigen-presenting capacity by increasing Rab4b-mediated endosomal recycling, informing studies to improve immune cell recognition (Krawczyk et al., 2007). In 3T3-L1 preadipocytes, Rab4b, together with Rab4a and Rab8a, mediates the recycling of the glucose transporter protein GLUT4 (Chen et al., 2012). It has also been demonstrated that Rab4b is involved in regulating endosomal circulation, which is essential for maintaining the spine and recirculating neurotransmitter receptors. At the same time, the neuron-specific effector of Rab4b, GRIP-associated protein-1 (GRASP-1), is a key component in coordinating the maturation of circulating endosomes in dendrites (Hoogenraad et al., 2010). This study not only further confirmed the impact of Rab4b on the cytotoxicity of *Gp*CDT but also conducted an initial exploration into the role of Rab4b within the vesicle trafficking of *Gp*CDT.

CdtB as an active subunit can induce significant cytotoxic and inflammatory responses in a wide range of cells, like THP-1 human macrophages (Chen et al., 2020), Human colorectal cancer cell line HCT116 cells, Human colonic epithelial cell line FHC cells (Gu et al., 2022), and Newborn pig tracheal epithelial cells (Yang et al., 2023). In this study, we first verified the connection between Rab4b and *Gp*CdtB, and first characterize the effects of Rab4b on *Gp*CDT-induced cytotoxicity in 3D4/21 cells. Furthermore, we employed indirect immunofluorescence to investigate the effect of Rab4b on *Gp*CDT vesicle trafficking in 3D4/21 cells. After cellular uptake, *Gp*CDT colocalized with early endosomes and the Golgi apparatus. Nevertheless, *Gp*CDT did not colocalize with the Golgi apparatus in Rab4b-KO cells. Thus, it is confirmed that the Rab4b protein interacts directly with *Gp*CDT, affecting its vesicle trafficking.

To further explore the mechanism by which Rab4b mediates *Gp*CDT vesicle trafficking, we performed transcriptomic analysis on 3D4/21 and Rab4b-KO cells after they were treated with *Gp*CDT. Through transcriptome sequencing, we found that the expression of 14 genes in the endocytosis pathway, in which Rab4b is located, was significantly altered, and only EEA1 was localized in early endosomes, similar to Rab4b. However, there are some functional differences between Rab4b and EEA1 (Jovic et al., 2010). Rab4b has been confirmed to participate in early endosomal sorting and endosomal circulation. EEA1, as an effector protein of Rab5, plays an important role in forming vesicles. It is a tethering molecule that provides directionality to vesicular transport from the plasma membrane to early endosomes (Adams and Wayne Vogl, 2017; McCaffrey et al., 2001; Rubino et al., 2000). Our study demonstrated that Rab4b can affect the expression of EEA1 in cells. After 3D4/21 cells were treated with *Gp*CDT, the level of the EEA1 protein changed significantly. However, Rab4b expression was not affected when EEA1 was knocked down. Moreover, the knockout of EEA1 inhibited the vesicle trafficking of *Gp*CDT and the uptake of *Gp*CDT by cells. We also tested the cellular uptake of *Gp*CDT in Rab4b-KO cells and reported that Rab4b knockout inhibited the uptake of *Gp*CDT by cells; however, green fluorescence of *GpCDT B* was still observed in Rab4b-KO cells. We speculated that the inhibition of *Gp*CDT uptake by Rab4b-KO cells was weaker than that by EEA1-KO cells because the knockout of Rab4b resulted in no upregulation of EEA1 expression in *Gp*CDT-treated cells, rather than a direct reduction in the expression of EEA1. Therefore, after binding to the cell membrane, *Gp*CDT can be separated from the cell membrane to form vesicles under the action of EEA1 and can be transported to early endosomes through vesicles. Subsequently, Rab4b mediates endosomal sorting in early endosomes, transports *Gp*CtdB to the Golgi apparatus, and circulates the *Gp*CDT receptor to the cell membrane. The knockout of Rab4b prevents the upregulation of the EEA1 protein, inhibits the formation of vesicles in which cells take up *Gp*CDT, and hinders the fusion of vesicles containing *Gp*CDT with early endosomes. Moreover, the Rab4b-mediated vesicle trafficking function is eliminated, resulting in a weakened toxic effect of *Gp*CDT on cells. Rab5 and its effector EEA1 mediate the delivery of internalized cargo molecules to the early endosomes. Cargoes can then be sorted at early endosomes.

Rab4b and EEA1 have been shown to assist bacterial infection. For example, *Clostridium difficile* binary toxins first bind to lipoprotein receptors (LSRs) stimulated by lipolysis on cell membranes (Hemmasi et al., 2015) and are then encased by clathrin to form vesicles that enter the cell and reach the early endosome (Papatheodorou et al., 2010). The interaction between EEA1 and Rab5 can regulate the occurrence of this process (Semerdjieva et al., 2008). Postentrant *Clostridium difficile* binary toxins are retrogradely transported from the endoplasmic reticulum through the Golgi apparatus via Rab4b-mediated vesicle trafficking into the cytoplasm (Schmidt et al., 2015), where they exert their toxic effects in the cytoplasm. In addition, *Pasteurella multocida* toxin (PMT) binds to low-density lipoprotein (LDL) receptor-associated protein 1 (LRP-1) on the cell membrane. It enters the cell (Schoellkopf et al., 2022), after which the vesicles coated with PMT fuse with early endosomes. Finally, the cells reach late endosomes, expand in the region, and then insert and translocate across the vesicle. The transfer of the catalytic domain into the cytoplasm is also influenced by EEA1 and Rab4b (Kubatzky, 2022; Repella et al., 2011). Additionally, Rab4b and EEA1 also play a crucial role in the virus’s infection. For example, during the process of Japanese Encephalitis Virus (JEV) entry, Rab4b and EEA1 play a crucial role in facilitating transport to early endosomes (Miao et al., 2025). Based on the cellular uptake and vesicle trafficking processes of *Clostridium difficile* binary toxins and *Pasteurella multicida* toxin, we hypothesized that *GpCDT* first binds to the receptor on the cell membrane to form a complex. Then EEA1 promotes the uptake of this complex by cells, leading to the formation of vesicles and their subsequent delivery to early endosomes. The complex subsequently disintegrates, and *GpCDT* interacts with Rab4b. The receptor of *GpCDT* returns to the cell membrane through early endosome sorting and binds to the next *Gp*CDT.

In summary, we conducted a systematic study to investigate the mechanism by which Rab4b contributes to the cytotoxicity of *Gp*CDT in 3D4/21 cells. Our findings demonstrate that Rab4b is essential for the transport of *Gp*CDT to endosomes. The mechanisms we identified contribute to a deeper understanding of how *Gp*CDT utilizes the Rab4b protein to facilitate infection, and may aid in the development of new strategies to control bacterial infections.

## 5 Conclusion

In conclusion, our investigation revealed, for the first time, that the host factor Rab4b enables the cellular uptake process of *Gp*CDT by upregulating EEA1 protein expression, thereby promoting the vesicle trafficking of *Gp*CDT in 3D4/21 cells and ultimately leading to the active subunit of *Gp*CDT-induced cytotoxicity. However, a limitation of this study is the lack of research on *Gp*CDT receptors and how RAB4B interacts with other Rab proteins to facilitate the transition of *Gp*CDT from early endosomes to late endosomes. In the future, we will continue to study the above issues.

## Data Availability

The data presented in the study are deposited in the SRA database, accession number PRJNA1332541.

## References

[ref1] Adams A. Wayne Vogl A. (2017). High resolution localization of Rab5, EEA1, and Nectin-3 to tubulobulbar complexes in the rat testis. Anat. Rec. 300, 1160–1170. doi: 10.1002/ar.23563 28176461

[ref2] Bergeland T. Haugen L. Landsverk O. J. Stenmark H. Bakke O. (2008). Cell-cycle-dependent binding kinetics for the early endosomal tethering factor EEA1. EMBO Rep. 9, 171–178. doi: 10.1038/sj.embor.7401152, PMID: 18188183 PMC2246413

[ref3] Boesze-Battaglia K. Dhingra A. Walker L. M. Zekavat A. Shenker B. J. (2020). Internalization and intoxication of human macrophages by the active subunit of the *Aggregatibacter actinomycetemcomitans* cytolethal distending toxin is dependent upon cellugyrin (synaptogyrin-2). Front. Immunol. 11:1262. doi: 10.3389/fimmu.2020.01262, PMID: 32655562 PMC7325893

[ref4] Brockmeier S. L. Loving C. L. Mullins M. A. Register K. B. Nicholson T. L. Wiseman B. S.. (2013). Virulence, transmission, and heterologous protection of four isolates of *Haemophilus parasuis*. Clin. Vaccine Immunol. 20, 1466–1472. doi: 10.1128/cvi.00168-13, PMID: 23885030 PMC3889593

[ref5] Brumell J. H. Scidmore M. A. (2007). Manipulation of rab GTPase function by intracellular bacterial pathogens. Microbiol. Mol. Biol. Rev. 71, 636–652. doi: 10.1128/mmbr.00023-07, PMID: 18063721 PMC2168649

[ref9001] Chen Y. Wang Y. Zhang J. Deng Y. Jiang L. Song E. (2012). Rab10 and myosin-Va mediate insulin-stimulated GLUT4 storage vesicle translocation in adipocytes. J Cell Biol 198:545–60. 22908308 10.1083/jcb.201111091 PMC3514028

[ref6] Chen M. X. Chen Y. Fu R. Mao G. Q. Liu S. Y. Shen T. B. (2020). Rab5a promotes cytolethal distending toxin B-induced cytotoxicity and inflammation. Infect. Immun. 88:e00132-20. doi: 10.1128/iai.00132-20, PMID: 32747601 PMC7504948

[ref7] Costa-Hurtado M. Barba-Vidal E. Maldonado J. Aragon V. (2020). Update on Glasser’s disease: how to control the disease under restrictive use of antimicrobials. Vet. Microbiol. 242:108595. doi: 10.1016/j.vetmic.2020.108595, PMID: 32122599

[ref8] Dixon S. D. Huynh M. M. Tamilselvam B. Spiegelman L. M. Son S. B. Eshraghi A.. (2015). Distinct roles for CdtA and CdtC during intoxication by cytolethal distending toxins. PLoS One 10:e0143977. doi: 10.1371/journal.pone.0143977, PMID: 26618479 PMC4664275

[ref9] Fouraux M. A. Deneka M. Ivan V. van der Heijden A. Raymackers J. van Suylekom D.. (2004). Rabip4′ is an effector of rab5 and rab4 and regulates transport through early endosomes. Mol. Biol. Cell 15, 611–624. doi: 10.1091/mbc.e03-05-0343, PMID: 14617813 PMC329268

[ref10] Gargi A. Reno M. Blanke S. R. (2012). Bacterial toxin modulation of the eukaryotic cell cycle: are all cytolethal distending toxins created equally? Front. Cell. Infect. Microbiol. 2:124. doi: 10.3389/fcimb.2012.00124, PMID: 23061054 PMC3465861

[ref11] Gu J. Lin Y. Wang Z. Pan Q. Cai G. He Q.. (2022). *Campylobacter jejuni* cytolethal distending toxin induces GSDME-dependent pyroptosis in colonic epithelial cells. Front. Cell. Infect. Microbiol. 12:853204. doi: 10.3389/fcimb.2022.853204, PMID: 35573789 PMC9093597

[ref12] He H. Dai F. Yu L. She X. Zhao Y. Jiang J.. (2002). Identification and characterization of nine novel human small GTPases showing variable expressions in liver cancer tissues. Gene Expr. 10, 231–242. doi: 10.3727/000000002783992406, PMID: 12450215 PMC5977521

[ref13] Hemmasi S. Czulkies B. A. Schorch B. Veit A. Aktories K. Papatheodorou P. (2015). Interaction of the *Clostridium difficile* binary toxin CDT and its host cell receptor, lipolysis-stimulated lipoprotein receptor (LSR). J. Biol. Chem. 290, 14031–14044. doi: 10.1074/jbc.M115.650523, PMID: 25882847 PMC4447975

[ref14] Hesping E. Boddey J. A. (2024). Whole-genome CRISPR screens to understand apicomplexan-host interactions. Mol. Microbiol. 121, 717–726. doi: 10.1111/mmi.15221, PMID: 38225194

[ref15] Hoogenraad C. C. Popa I. Futai K. Martinez-Sanchez E. Wulf P. S. van Vlijmen T.. (2010). Neuron specific Rab4 effector GRASP-1 coordinates membrane specialization and maturation of recycling endosomes. PLoS Biol. 8:e1000283. doi: 10.1371/journal.pbio.1000283 20098723 PMC2808209

[ref16] Hu X. Wang Y. (2024). Protocol to identify receptors of secreted proteins through CRISPR-Cas9 whole-genome screening technology. STAR Protoc. 5:103315. doi: 10.1016/j.xpro.2024.103315, PMID: 39277866 PMC11419825

[ref17] Huhn G. R. 3rd Sparkes C. Silva I. Reyes C. Perez G. Khondker F.. (2022). Acid-induced disassembly of the *Haemophilus ducreyi* cytolethal distending toxin. Biochem. Biophys. Res. Commun. 636, 57–63. doi: 10.1016/j.bbrc.2022.10.068 36332483

[ref18] Jordens I. Marsman M. Kuijl C. Neefjes J. (2005). Rab proteins, connecting transport and vesicle fusion. Traffic 6, 1070–1077. doi: 10.1111/j.1600-0854.2005.00336.x, PMID: 16262719

[ref19] Jovic M. Sharma M. Rahajeng J. Caplan S. (2010). The early endosome: a busy sorting station for proteins at the crossroads. Histol. Histopathol. 25, 99–112. doi: 10.14670/hh-25.99, PMID: 19924646 PMC2810677

[ref20] Kaddai V. Gonzalez T. Keslair F. Gremeaux T. Bonnafous S. Gugenheim J.. (2009). Rab4b is a small GTPase involved in the control of the glucose transporter GLUT4 localization in adipocyte. PLoS One 4:e5257. doi: 10.1371/journal.pone.0005257, PMID: 19590752 PMC2707114

[ref21] Kailoo S. Shreya Kumar Y. (2021). Cytolethal distending toxin: from genotoxin to a potential biomarker and anti-tumor target. World J. Microbiol. Biotechnol. 37:150. doi: 10.1007/s11274-021-03117-z 34379213

[ref22] Kalin S. Buser D. P. Spiess M. (2016). A fresh look at the function of Rabaptin5 on endosomes. Small GTPases 7, 34–37. doi: 10.1080/21541248.2016.1140616, PMID: 26940354 PMC4905272

[ref23] Krawczyk M. Leimgruber E. Seguin-Estevez Q. Dunand-Sauthier I. Barras E. Reith W. (2007). Expression of RAB4B, a protein governing endocytic recycling, is co-regulated with MHC class II genes. Nucleic Acids Res. 35, 595–605. doi: 10.1093/nar/gkl980, PMID: 17175541 PMC1802633

[ref24] Kubatzky K. F. (2022). *Pasteurella multocida* toxin—lessons learned from a mitogenic toxin. Front. Immunol. 13:1058905. doi: 10.3389/fimmu.2022.1058905, PMID: 36591313 PMC9800868

[ref25] Mao W. Wang Z. Wen S. Lin Y. Gu J. Sun J.. (2023). LRRC8A promotes *Glaesserella parasuis* cytolethal distending toxin-induced p53-dependent apoptosis in NPTr cells. Virulence 14:2287339. doi: 10.1080/21505594.2023.2287339, PMID: 38018865 PMC10732598

[ref26] McCaffrey M. W. Bielli A. Cantalupo G. Mora S. Roberti V. Santillo M.. (2001). Rab4 affects both recycling and degradative endosomal trafficking. FEBS Lett. 495, 21–30. doi: 10.1016/s0014-5793(01)02359-6, PMID: 11322941

[ref27] Miao C. Zhao Q. Zhang Y. T. Luo S. Q. Han X. Wen Y.. (2025). RAB4B and Japanese encephalitis virus E protein interaction is essential for viral entry in early endosomes. Int. J. Biol. Macromol. 306:141452. doi: 10.1016/j.ijbiomac.2025.141452, PMID: 40020812

[ref28] Mills I. G. Jones A. T. Clague M. J. (1998). Involvement of the endosomal autoantigen EEA1 in homotypic fusion of early endosomes. Curr. Biol. 8, 881–884. doi: 10.1016/s0960-9822(07)00351-x, PMID: 9705936

[ref29] Mishra A. Eathiraj S. Corvera S. Lambright D. G. (2010). Structural basis for Rab GTPase recognition and endosome tethering by the C2H2 zinc finger of early endosomal autoantigen 1 (EEA1). Proc. Natl. Acad. Sci. U.S.A. 107, 10866–10871. doi: 10.1073/pnas.1000843107, PMID: 20534488 PMC2890723

[ref30] Mu F. T. Callaghan J. M. Steele-Mortimer O. Stenmark H. Parton R. G. Campbell P. L.. (1995). EEA1, an early endosome-associated protein. EEA1 is a conserved alpha-helical peripheral membrane protein flanked by cysteine “fingers” and contains a calmodulin-binding IQ motif. J. Biol. Chem. 270, 13503–13511. doi: 10.1074/jbc.270.22.13503, PMID: 7768953

[ref31] Ni H. B. Gong Q. L. Zhao Q. Li X. Y. Zhang X. X. (2020). Prevalence of *Haemophilus parasuis* “*Glaesserella parasuis*” in pigs in China: a systematic review and meta-analysis. Prev. Vet. Med. 182:105083. doi: 10.1016/j.prevetmed.2020.105083, PMID: 32652336

[ref32] Papatheodorou P. Zamboglou C. Genisyuerek S. Guttenberg G. Aktories K. (2010). Clostridial glucosylating toxins enter cells via clathrin-mediated endocytosis. PLoS One 5:e10673. doi: 10.1371/journal.pone.0010673, PMID: 20498856 PMC2871790

[ref33] Pereira-Leal J. B. Seabra M. C. (2001). Evolution of the Rab family of small GTP-binding proteins. J. Mol. Biol. 313, 889–901. doi: 10.1006/jmbi.2001.5072, PMID: 11697911

[ref34] Perrin L. Lacas-Gervais S. Gilleron J. Ceppo F. Prodon F. Benmerah A.. (2013). Rab4b controls an early endosome sorting event by interacting with the gamma-subunit of the clathrin adaptor complex 1. J. Cell Sci. 126, 4950–4962. doi: 10.1242/jcs.130575 24006255

[ref35] Pons B. J. Vignard J. Mirey G. (2019). Cytolethal distending toxin subunit B: a review of structure-function relationship. Toxins 11:595. doi: 10.3390/toxins11100595, PMID: 31614800 PMC6832162

[ref36] Repella T. L. Ho M. Chong T. P. Bannai Y. Wilson B. A. (2011). Arf6-dependent intracellular trafficking of *Pasteurella multocida* toxin and pH-dependent translocation from late endosomes. Toxins 3, 218–241. doi: 10.3390/toxins3030218, PMID: 22053287 PMC3202820

[ref37] Rubino M. Miaczynska M. Lippe R. Zerial M. (2000). Selective membrane recruitment of EEA1 suggests a role in directional transport of clathrin-coated vesicles to early endosomes. J. Biol. Chem. 275, 3745–3748. doi: 10.1074/jbc.275.6.3745, PMID: 10660521

[ref38] Schmidt G. Papatheodorou P. Aktories K. (2015). Novel receptors for bacterial protein toxins. Curr. Opin. Microbiol. 23, 55–61. doi: 10.1016/j.mib.2014.11.003, PMID: 25461573

[ref39] Schoellkopf J. Mueller T. Hippchen L. Mueller T. Reuten R. Backofen R.. (2022). Genome wide CRISPR screen for *Pasteurella multocida* toxin (PMT) binding proteins reveals LDL receptor related protein 1 (LRP1) as crucial cellular receptor. PLoS Pathog. 18:e1010781. doi: 10.1371/journal.ppat.1010781, PMID: 36516199 PMC9797058

[ref40] Scuron M. D. Boesze-Battaglia K. Dlakic M. Shenker B. J. (2016). The cytolethal distending toxin contributes to microbial virulence and disease pathogenesis by acting as a tri-perditious toxin. Front. Cell. Infect. Microbiol. 6:168. doi: 10.3389/fcimb.2016.00168, PMID: 27995094 PMC5136569

[ref41] Semerdjieva S. Shortt B. Maxwell E. Singh S. Fonarev P. Hansen J.. (2008). Coordinated regulation of AP2 uncoating from clathrin-coated vesicles by rab5 and hRME-6. J. Cell Biol. 183, 499–511. doi: 10.1083/jcb.200806016, PMID: 18981233 PMC2575790

[ref42] Shenker B. J. Boesze-Battaglia K. Scuron M. D. Walker L. P. Zekavat A. Dlakic M. (2016). The toxicity of the *Aggregatibacter actinomycetemcomitans* cytolethal distending toxin correlates with its phosphatidylinositol-3,4,5-triphosphate phosphatase activity. Cell. Microbiol. 18, 223–243. doi: 10.1111/cmi.12497 26247396

[ref43] Simonsen A. Lippé R. Christoforidis S. Gaullier J. M. Brech A. Callaghan J.. (1998). EEA1 links PI(3)K function to Rab5 regulation of endosome fusion. Nature 394, 494–498. doi: 10.1038/28879, PMID: 9697774

[ref44] Somsel Rodman J. Wandinger-Ness A. (2000). Rab GTPases coordinate endocytosis. J. Cell Sci. 113, 183–192. doi: 10.1242/jcs.113.2.183 10633070

[ref45] Spano S. Galan J. E. (2018). Taking control: hijacking of Rab GTPases by intracellular bacterial pathogens. Small GTPases 9, 182–191. doi: 10.1080/21541248.2017.1336192, PMID: 28632996 PMC5902217

[ref46] Stenmark H. Aasland R. Toh B. H. D’Arrigo A. (1996). Endosomal localization of the autoantigen EEA1 is mediated by a zinc-binding FYVE finger. J. Biol. Chem. 271, 24048–24054. doi: 10.1074/jbc.271.39.24048, PMID: 8798641

[ref47] Yang Z. Zhang Y. Du S. Zhao Q. Huang X. Wu R.. (2023). Upregulation of occludin by cytolethal distending toxin facilitates *Glaesserella parasuis* adhesion to respiratory tract cells. Infect. Immun. 91:e0035123. doi: 10.1128/iai.00351-23, PMID: 37930004 PMC10715221

[ref48] Yeh J. Y. Lin H. J. Kuo C. J. Feng C. L. Chou C. H. Lin C. D.. (2020). *Campylobacter jejuni* cytolethal distending toxin C exploits lipid rafts to mitigate *Helicobacter pylori*-induced pathogenesis. Front. Cell Dev. Biol. 8:617419. doi: 10.3389/fcell.2020.617419, PMID: 33708766 PMC7940356

[ref49] Zhang Y. Yang Z. Dai K. Hu B. Xu S. Wang Y.. (2024). Rab4b promotes cytolethal distending toxin from *Glaesserella parasuis*-induced cytotoxicity in PK-15 cells. Toxins 16:407. doi: 10.3390/toxins16090407, PMID: 39330865 PMC11435814

