# Supplementary material for: Host factor Rab4b mediates internalization and intoxication of 3D4/21 cells by the active subunit of the Glaesserella parasuis cytolethal distending toxin via influencing EEA1 expression
Source: Front Microbiol. 2025 Oct 31;16:1660176. doi: 10.3389/fmicb.2025.1660176 (PMC12615497; doi:10.3389/fmicb.2025.1660176)
Supplement: Supplementary file 1 [file Table_1.docx]

**Supplementary Tables**

Table S1 Schedule of quality data output

| Sample name | Raw reads | Clean reads | Clean bases | Error rate(%) | Q20(%) | Q30(%) | GC content(%) |
| --- | --- | --- | --- | --- | --- | --- | --- |
| KO_*Gp*CDT_1 | 56040086 | 54830986 | 8.22G | 0.03 | 97.81 | 94.1 | 52.48 |
| KO_*Gp*CDT_2 | 50205960 | 49095368 | 7.36G | 0.02 | 97.91 | 94.35 | 53.19 |
| KO_*Gp*CDT_3 | 49769030 | 48744676 | 7.31G | 0.03 | 97.82 | 94.05 | 51.63 |
| WT_*Gp*CDT_1 | 46748922 | 45978008 | 6.90G | 0.02 | 97.93 | 94.43 | 51.96 |
| WT_*Gp*CDT_2 | 62899210 | 61475760 | 9.22G | 0.03 | 97.54 | 93.52 | 52.07 |
| WT_*Gp*CDT_3 | 47529658 | 46364170 | 6.95G | 0.03 | 97.77 | 94.1 | 53.34 |
