# Supplementary material for: Host factor Rab4b mediates internalization and intoxication of 3D4/21 cells by the active subunit of the Glaesserella parasuis cytolethal distending toxin via influencing EEA1 expression
Source: Front Microbiol. 2025 Oct 31;16:1660176. doi: 10.3389/fmicb.2025.1660176 (PMC12615497; doi:10.3389/fmicb.2025.1660176)
Supplement: Supplementary file 2 [file Table_2.docx]

**Supplementary Tables**

Table S2 Primers required to validate transcriptome sequencing result

| Gene | Primer direction | Sequence (5’–3’) | size (bp) |
| --- | --- | --- | --- |
| 396655 | 396655-F  396655-R | CAGAACCGATGGAGCTTGACGAC  GCAGGTTCTCTGTGGTCTGAAGTC | 107bp |
| 100049703 | 100049703-F  100049703-R | GAGCGTAGACAAGGAGATGC  TCCGACTGAAGAGCGAAC | 239bp |
| 396621 | 396621-F  396621-R | GCCTGGGTGAAGAATGAACTA  GATTGTGGTGGGCAAAGTAGA | 208bp |
| 494019 | 494019-F  494019-R | CGGCACTACTGATAAGGATGG  TGCTCAACAGCTCGGGATG | 163bp |
| 396609 | 396609-F  396609-R | CTTCTACGACGATGCCCTCAACG  GCCAGGTTCAGGGTCATGCTCTG | 105bp |
| 100511937 | 100511937-F  100511937-R | CCAAGAGTTCCGTAAACAGTGCTA  CTGCATGTAATGGACTGCCTCT | 196bp |
| 396880 | 396880-F  396880-R | CACCATCATGATAGCATCTGT  CCTGAGTCATCACACTTCCT | 287bp |
| 100322893 | 100322893-F  100322893-R | CCACACCCAAAGTCCTCACT  CACTGCTGCTTT CCGTAACA | 220bp |
| 100515607 | 100515607-F  100515607-R | AAGTGCTGCGCAGTATTAGCA  TCTGGACCCACAAAGCCAAT | 107bp |
| 100126276 | 100126276-F  100126276-R | TCCTGACAACCCCGAAAGTC  ACGAAGTTTGCCCCCGA | 98bp |
| 100513811 | 100513811-F  100513811-R | GAAGCTGCTGGGCAAAGG  CGGTCGTGGGTCTGGAA | 207bp |
| 100155841 | 100155841-F  100155841-R | AGAGTCTCTGTTGACTTGCTGG  GGCTTGGGCTGCTGTAATTC | 72bp |
